# The role of oxidative stress in aortic dissection: a potential therapeutic target

**DOI:** 10.3389/fcvm.2024.1410477

**Published:** 2024-07-12

**Authors:** Shengnan Xu, Xueyu Han, Xiukun Wang, Yi Yu, Chuan Qu, Xin Liu, Bo Yang

**Affiliations:** ^1^ Department of Cardiology, Renmin Hospital of Wuhan University, Wuhan, China; ^2^ Cardiovascular Research Institute, Wuhan University, Wuhan, China; ^3^ Hubei Key Laboratory of Cardiology, Wuhan University, Wuhan, China

**Keywords:** aortic dissection, oxidative stress, vascular smooth muscle cells, extracellular matrix, inflammatory cells, endothelial cells

## Abstract

The incidence of aortic dissection (AD) is steadily increasing, driven by the rising prevalence of chronic conditions such as hypertension and the global aging of the population. Oxidative stress emerges as a pivotal pathophysiological mechanism contributing to the progression of AD. Oxidative stress triggers apoptosis in vascular smooth muscle cells, reshapes the extracellular matrix (ECM), and governs ECM degradation and remodeling, subsequently impacting aortic compliance. Furthermore, oxidative stress not only facilitates the infiltration of macrophages and mononuclear lymphocytes but also disrupts the integral structure and functionality of endothelial cells, thereby inducing endothelial cell dysfunction and furthering the degeneration of the middle layer of the aortic wall. Investigating antioxidants holds promise as a therapeutic avenue for addressing AD.

## 1 Introduction

Aortic dissection (AD) occurs when there is a breach in the lining of the arterial wall and blood enters the arterial wall through the breach to form a hematoma, which further strips the intima and media of the aorta. This condition results in the separation and longitudinal expansion of the aortic media, creating a division within the aortic wall characterized by the presence of an intimal valve. The newly torn diseased lumen is called the false lumen and the original lumen with normal blood flow is called the true lumen. Anatomically, acute AD (AAD) can be classified based on the location of the intimal tear or the extent of aortic involvement, irrespective of the specific tear location. Stanford's classification further categorizes dissections into those involving the ascending aorta (Type A) or not (Type B) (1). The incidence of AAD stands at approximately 2.6–3.5 cases per 100,000 individuals per year, which is half the incidence rate observed for symptomatic aortic aneurysms (2, 3). Without prompt surgical intervention, AD rupture leads to a mortality rate exceeding 80%. Recent advances in treatment have significantly reduced the overall in-hospital mortality rate for Type A AAD. However, studies on Type B AAD have not yielded equally effective outcomes (4). Epidemiological investigations into AD may underestimate its actual incidence due to the absence of worldwide and prospective population-based studies. Hypertension stands as the most prevalent risk factor for AAD (5). In recent years, the prevalence of AD is expected to rise significantly, owing to the increasing prevalence of chronic diseases such as hypertension and the global aging of the population (2).

Oxidative stress refers to an imbalance between the production of reactive oxygen species (ROS) and endogenous antioxidant defense mechanisms. ROS encompasses various reactive oxygen-containing chemicals, including superoxide ($O_{2}^{\cdot -}$) and hydroxyl ($HO^{\cdot }$) radicals, as well as non-radical molecules such as hydrogen peroxide (*H*_2_*O*_2_). These molecules are predominantly generated during metabolic processes in mitochondria, peroxisomes, and the endoplasmic reticulum (6). Among these organelles, mitochondria are recognized as the primary source of ROS. While the human body can maintain oxidative/antioxidative equilibrium under normal conditions, this balance may be disrupted in pathophysiological conditions such as AD. Elevated ROS levels can lead to cellular dysfunction, protein and lipid peroxidation, DNA damage, and ultimately irreversible cellular injury and apoptosis (6). Oxidative stress has been implicated in the pathogenesis of numerous chronic diseases, including cardiovascular diseases (7–9). Numerous endogenous enzyme systems that regulate oxidative stress in AD have been scrutinized through clinical investigations and animal experiments spanning several decades (10, 11). Oxidative stress is intimately linked to the progression of AD, and a comprehensive understanding of its role in AD's development can pave the way for potential diagnostic, prognostic, and therapeutic applications in this condition.

## 2 ROS generation

ROS are typically generated through a series of reactions that result in the production of superoxide. Superoxide undergoes rapid spontaneous conversion into *H*_2_*O*_2_, or catalysis by superoxide dismutase (SOD) to form *H*_2_*O*_2_. Subsequently, a cascade of reactions ensues, which includes the interaction of superoxides with nitric oxide (NO) to yield peroxynitrite (ONOO^−^) and the conversion of *H*_2_*O*_2_ into water, eventually leading to the generation of hydroxyl radicals (12).

A significant source of ROS emerges from the end substrate of the respiratory chain, situated within the inner mitochondrial membrane. Here, a fraction of *O*_2_ undergoes reduction during the transfer of electrons from the mitochondrial electron transport chain complexes, yielding $O_{2}^{\cdot -}$ or *H*_2_*O*_2_. The most noteworthy among these ROS is $O_{2}^{\cdot -}$, which serves as the principal precursor for the majority of ROS (12). Besides, there are numerous enzymatic systems can generate ROS, such as nicotinamide adenine dinucleotide (NADH)/nicotinamide adenine dinucleotide phosphate (NADPH) oxidase (NOX), xanthine oxidase (XO), and uncoupled endothelial nitric oxide synthase (eNOS) (13). Another significant ROS source is NOX, which is an enzymatic complex (14).

NOX functions as a crucial coenzyme within cells, facilitating the catalytic transfer of electrons from NADPH to *O*_2_, resulting in $O_{2}^{\cdot -}$ production. NOX systems have become central players in vascular diseases and heart-related conditions. They are associated with the contraction, proliferation, apoptosis, and inflammation of vascular smooth muscle cells (VSMCs) (15, 16). The catalytic subunit gp91phox of NOX is commonly referred to as NOX2. NOX1, NOX2, NOX4, and NOX5 are expressed and functionally active in human blood vessel cells, and they are implicated in altering redox states in vascular diseases (16). NOX2 exhibits limited expression in endothelial cells, and elevated expression of NOX2 can augment vascular NOX activity and oxidative stress (17). XO in the purine catabolic pathway is accompanied by the production of the reaction by-product (18). The other source of ROS in metabolic and vascular diseases is the uncoupling of eNOS. eNOS is predominantly distributed in endothelial cells and exists as a homodimer. It utilizes L-arginine (L-Arg) and oxygen as substrates to synthesize NO and citrulline. Tetrahydrobiopterin (BH_4_) serves as an indispensable cofactor for eNOS. In cases of eNOS uncoupling, $O_{2}^{\cdot -}$ is produced instead of NO, and the generated $O_{2}^{\cdot -}$ subsequently reacts with NO to form ONOO^−^ (19).

**Figure 1:**
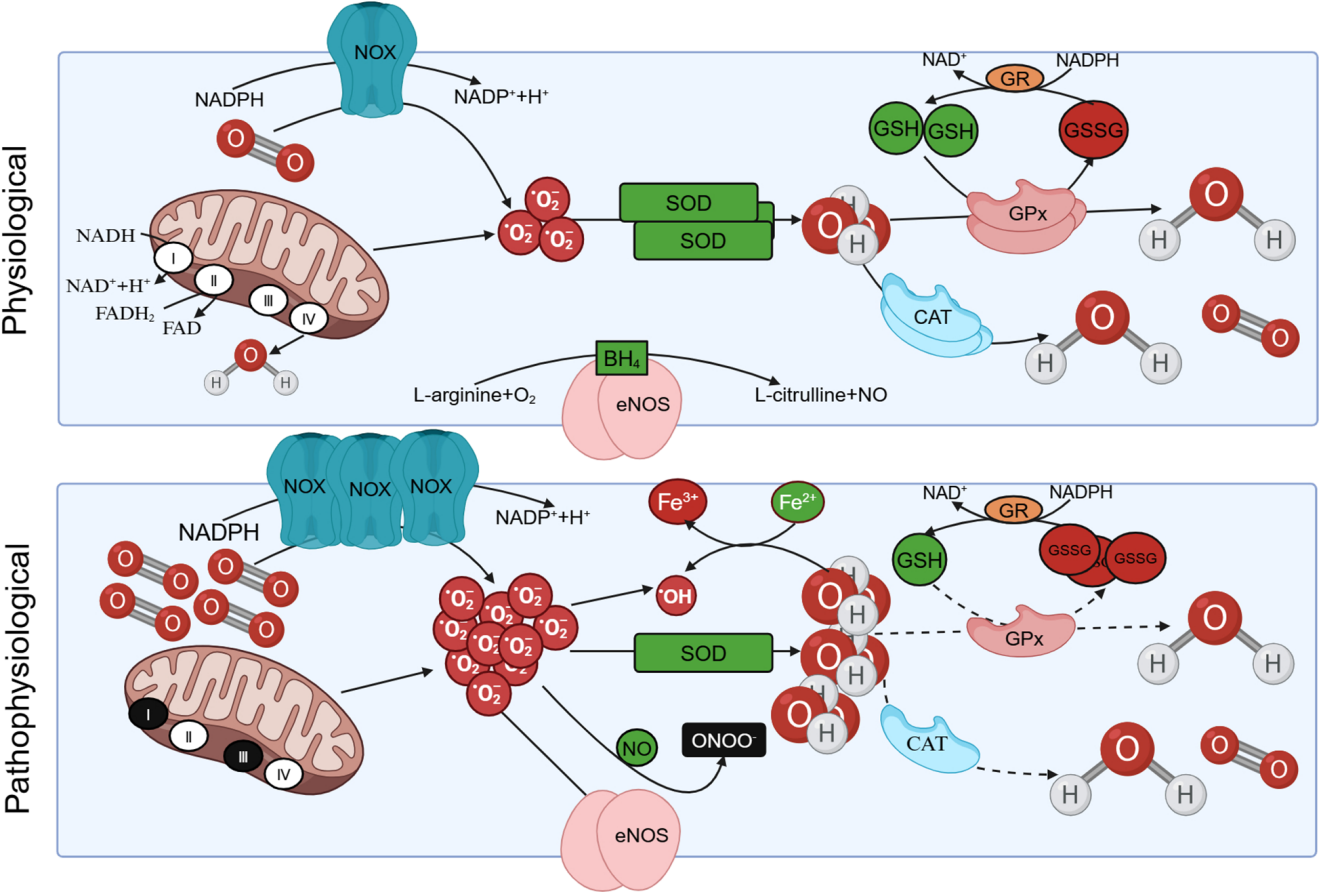
The production of reactive oxygen species (ROS) under physiological and pathological conditions. Under physiological conditions, protease complex Ⅰ and Ⅲ in the respiratory chain of mitochondrial intima can reduce oxygen to $O_{2}^{\cdot -}$ with the oxidation of NADH. Triphosphopyridine nucleotide (NADPH) oxidase (NOX) also facilitates the catalytic transfer of electrons from NADPH to *O*_2_, resulting in the production of $O_{2}^{\cdot -}$. The $O_{2}^{\cdot -}$ can be eliminated by oxidation reactions mediated by antioxidants such as superoxide dismutase (SOD), yielding *O*_2_ and *H*_2_*O*_2_. Subsequently, *H*_2_*O*_2_ can undergo decomposition into *O*_2_ and H_2_O through the catalytic action of catalase (CAT), or react with GSH in the presence of glutathione peroxidase (GPx), resulting in the formation of oxidized glutathione (GSSG). The conversion of GSSG back to GSH is accomplished by the enzyme glutathione reductase (GR). In pathological conditions, $O_{2}^{\cdot -}$ is overproduced, the intracellular antioxidant system is unbalanced, resulting in oxidative stress. Besides, eNOS utilizes L-arginine (L-Arg) and oxygen as substrates to synthesize NO and L-citrulline. Tetrahydrobiopterin (BH4) serves as an indispensable cofactor for eNOS. eNOS undergoes uncoupling and $O_{2}^{\cdot -}$ is produced instead of NO, and the generated $O_{2}^{\cdot -}$ subsequently reacts with NO to form ONOO^–^ under pathological conditions.

Under normal conditions, the elimination of ROS is mediated by antioxidant systems, which can be categorized into enzyme and non-enzyme systems. The enzyme system comprises SOD, catalase (CAT), and glutathione peroxidase (GPx). The non-enzyme system primarily includes reduced glutathione (GSH), vitamin C/E, and other compounds (8). SOD facilitates the dismutation of superoxide anion radicals, yielding *O*_2_ and *H*_2_*O*_2_. Subsequently, *H*_2_*O*_2_ can undergo decomposition into *O*_2_ and H_2_O through the catalytic action of CAT or react with GSH in the presence of GPx, resulting in the formation of oxidized glutathione (GSSG) and water. The conversion of GSSG back to GSH is accomplished by the enzyme glutathione reductase (GR) (18). In pathological states, excessive ROS production and an imbalance in intracellular antioxidant systems give rise to oxidative stress, leading to damage in human tissues (Figure 1).

## 3 ROS-mediated signaling pathway

The physiological consequences of oxidative stress primarily stem from its capacity to modulate diverse signaling pathways, including the nuclear factor κB (NF-κB), mitogen-activated protein kinase (MAPK), Kelch-like ECH-associated protein 1/nuclear factor E2-related factor 2 (Keap1/Nrf2), and AMP-activated protein kinase (AMPK) pathways. On the one hand, an overabundance of ROS activates multiple transcription factors, such as NF-κB and Nrf2, thereby inducing the synthesis of proteins that govern inflammation, cellular transformation, cell viability, proliferation, invasion, angiogenesis, and metastasis. On the other hand, it triggers distinct cellular death pathways, such as apoptosis, necrosis, and autophagy.

### 3.1 NF-κB pathway

The activation of the NF-κB pathway in cells is a significant contributor to inflammation. Dysregulation of NF-κB activity is associated with inflammation-related diseases and cancer (20, 21). NF-κB can promote the transcription of various inflammatory response genes, including those of tumor necrosis factor-α (TNF-α) and interleukin (IL)-1β (22). The cellular ROS levels influence the NF-κB activity level, illustrating the interdependence between NF-κB and ROS in gene transcription (23). The P21-activated kinase (PAK) serves as a crucial regulator of actin dynamics, cell proliferation, and cell survival. PAK modulates NF-κB activation by regulating cellular sensitivity to ROS (24).

The NF-κB family includes five related transcription factors, and common NF-κB protein is referred to homologous or heterodimers composed of p65/p50 subunit (22). It stays inactive in the cytoplasm by binding to the inhibitor of NF-κB (IκB) to form a trimeric complex (22). Two distinct signaling pathways govern the activation of NF-κB: the classical and the non-classical pathways. ROS regulate the classical NF-κB pathway through the activation of TNF-α (25–27), Toll-like receptor family (TLRs) (28–31), and interleukin cytokine receptors. TNF-α can activate the NF-κB signaling pathway and induce activation of caspase-8, which leads to ROS production and promotes apoptosis (26). In the human genome, 10 TLRs (TLR1–TLR10) have been identified (32). Among these receptors, TLR4 serves as the principal lipopolysaccharide (LPS) receptor. When bacterial LPS binds to TLR4, the NF-κB signaling pathway is initiated through intermediaries such as myeloid differentiation factor 88 (MYD88) (33). ROS regulates NF-κB-dependent transcription through its involvement in TLR4-mediated early cellular responses (34). Knockdown of TLR4 inhibits both the NF-κB pathway and ROS production (29). ROS plays a bidirectional role in NF-κB activation. In the initial stages, ROS induces NF-κB activation through both classical and non-classical pathways. Subsequently, ROS inhibits NF-κB activation (35). Both pathways entail the activation of the IκB kinase (IKK) complex, resulting in the phosphorylation and dissociation of the IκB protein from the trimer. IKK represents another crucial target of ROS to influence NF-κB signaling (23). *H*_2_*O*_2_ can deactivate IKK, which may be mediated through oxidation of IKKβ at cysteine 179 by ROS. IKKβ becomes S-glutathionylated upon exposure to ROS, thereby deactivating kinase activity and leading to reduced NF-κB signaling (36).

### 3.2 MAPK pathway

The MAPK signaling pathway constitutes a pivotal component within the eukaryotic signal transmission network, serving as a primary conduit for cellular processes such as proliferation, differentiation, apoptosis, and stress response under both normal and pathological conditions (37). MAPKs are further categorized into four distinct subfamilies, namely extracellular signal-regulated kinase (ERK), p38, Jun N-terminal kinase (JNK), and ERK5 (38). ERK1/2 have been extensively researched, and the ERK1/2 signaling pathway plays a role in both apoptosis induced by oxidative damage and safeguarding against such apoptosis (38, 39). This pathway proceeds through the tertiary kinase cascade of MAPK, with Ras serving as the activating protein upstream. The three primary subtypes of small GTPase p21 Ras are N-Ras, Kirsten Ras (Ki-Ras), and Harvey Ras (Ha-Ras), which plays a direct role in regulating cellular redox status (40). Ha-Ras potentially serves as a direct receptor of ROS, triggering the NOX system to generate more ROS, whereas Ki-Ras facilitates ROS clearance through the activation of the ERK1/2-dependent pathway (41). Following oxidative stress, Ha-Ras augmentation leads to increased apoptosis among cells. Conversely, Ki-Ras shields against *H*_2_*O*_2_-induced apoptosis by reducing intracellular ROS levels and counteracting the actions of Ha-Ras (40, 41).

The JNK signal transduction pathway represents a vital branch within the MAPK pathway. ROS serves as an upstream signaling molecule for JNK, functioning as a secondary messenger within the JNK signaling pathway (42). Furthermore, ROS regulates various signaling proteins, including apoptosis signal regulating kinase-1 (ASK1) and Src kinase, thereby facilitating JNK activation through apoptosis signals (43–45). Deletion of the Src gene and inhibition of Src kinase activity markedly suppress JNK activation in the ROS-mediated JNK signaling pathway, with no discernible impact on ERK and p38 activation (46). Moreover, ROS is a crucial link between the NF-κB and JNK signaling pathway. The NF-κB and JNK signaling pathways share numerous upstream molecules, permitting mutual influence via ROS (47). NF-κB, by inhibiting ROS formation and consistently activating the JNK cascade, participates in survival signaling, effectively counteracting programmed cell death induced by the TNF-α family of receptors (47).

### 3.3 Nrf2 pathway

Nrf2 assumes a critical role by activating the body's antioxidant response through binding to antioxidant response elements (ARE) in the promoter regions of numerous genes responsible for cellular protection (48). Nrf2 governs four genes associated with NADPH synthesis and orchestrates the expression of key enzymes involved in GSH production and utilization (49). A deficiency in Nrf2 results in heightened NOX2 activity (50). Moreover, Nrf2 controls several redox protein family auxiliary proteins, such as thioredoxin (Trx) (49). Trx1 fosters oxidative phosphorylation and tricarboxylic acid (TCA) cycling in cardiomyocytes via Nrf2 activation, which concurrently stimulates Trx1 expression (51). Beyond these direct effects on redox status, Nrf2 also modulates the expression of genes implicated in cellular defense, thereby inhibiting redox cycle reactions and diminishing GSH consumption (49). However, excessive Nrf2 activation may contribute to the onset and progression of vascular diseases (52).

Keap1 functions as an interacting partner of Nrf2, binding with Nrf2 to facilitate its degradation and exert a negative regulatory influence on Nrf2 activity. The Keap1/Nrf2/ARE pathway modulates cellular redox equilibrium to uphold cell homeostasis (53). Under normal conditions, Keap1 complex-mediated ubiquitination degrades Nrf2, and when confronted with oxidative stress, Keap1 complex activity is inhibited and the Nrf2/ARE pathway is activated. This activation results in the induction of an array of cytoprotective genes, such as those of NAD(P)H quinone dehydrogenase 1 (NQO1), heme oxygenase 1 (HO1), GPx, GR, and SOD (54, 55). Furthermore, the Keap1/Nrf2/ARE pathway exerts regulatory control over GSH levels by upregulating essential enzymes involved in GSH synthesis (54). Nrf2 interacts with the NF-κB signaling pathway, and its depletion enhances NF-κB activity, thereby augmenting cytokine production. Conversely, NF-κB regulates both the transcription and activity of Nrf2 (56). Central to the Nrf2-mediated inhibition of NF-κB is HO1. HO1 stands as a critical antioxidant enzyme. HO1 exhibits beneficial effects by preventing oxidative damage, regulating apoptosis, modulating inflammation, and promoting angiogenesis (48). This elevated activity of HO1 effectively hampers the transcription of NF-κB-mediated adhesion molecules (56).

### 3.4 AMPK pathway

AMPK, a serine/threonine kinase, plays a critical role in regulating energy homeostasis, metabolism, and mitochondrial equilibrium (57). AMPK is a heterotrimeric complex comprising a catalytic subunit *α* and two regulatory subunits β and γ. The binding of AMP to the γ subunit induces allosteric activation of AMPK, whereas ATP can inhibit this activation (58). ROS disrupt oxidative metabolism, but AMPK activation helps maintain cellular oxidative metabolism stability (59). AMPK also modulates inflammatory responses by regulating the JNK and NF-κB signaling pathways, protecting against ROS-induced apoptosis. Chronic AMPK activation diminishes JNK activation and inhibition of the JNK signaling pathway reduces NF-κB activity (60). The AMPK agonist metformin can curb both ROS production and the JNK/NF-κB signaling cascade (60).

The protective effects of the AMPK signaling pathway on cells are associated with the regulation of mammalian target of rapamycin (mTOR) and sirtuins (SIRT). mTOR is an important regulator of cellular growth and proliferation. Excessive ROS can negatively regulate mTORC1, and AMPK plays a pivotal role in mediating autophagy by suppressing mTOR (61). Within the SIRT protein family, SIRT3 is closely associated with mitochondrial function and oxidative stress. In mice, SIRT3 inhibits the generation of ROS and alleviates oxidative stress and cardiac hypertrophy by activating the AMPK pathway (62). SIRT3 deficiency fosters the development of AD through reducing the anti-ROS effect while increasing VSMC apoptosis and vascular inflammation (63).

## 4 Oxidative stress and AD

The pathophysiology of AD is multifaceted, and impaired aortic integrity is a fundamental mechanism in the pathology of AD, which results from the inherent instability of the aortic wall (e.g., due to inherited connective tissue diseases) or acquired conditions (e.g., atherosclerotic degeneration associated with aging) (64). Patients with genetic disorders including Marfan syndrome, Lloyd-Dietz syndrome, and vascular Ehlers-Danlos syndrome often develop AD called hereditary thoracic aortic disease (HTAD) (65, 66). Currently, there are two main modes of construction of AD mouse models: genetically modified models (e.g., genetic manipulation of fibrillin-1 or transforming growth factor β receptor 1/transforming growth factor β receptor 2 mutations) (67, 68) and chemically induced models [e.g., administration of Ang II, calcium chloride, elastase, or β-aminopropionitrile (BAPN)] (69, 70). One of the limitations of the mouse model is the difference in the number of elastic layers in the media, 7 layers in mice vs. 50 layers in humans, which may severely affect the mechanical relevance of the model. Nevertheless, these models can provide valuable data on the initiation of AD, which can help in understanding human AD (68). Large animal models regarding AD are mainly constructed surgically and can be used for the study of disease progression and treatment modalities, but they are technically demanding, can only study surgically induce traumatic entrapment, are unsuitable for the study of AD pathogenesis, and are not as reproducible as small animal models (71, 72).

**Figure 2:**
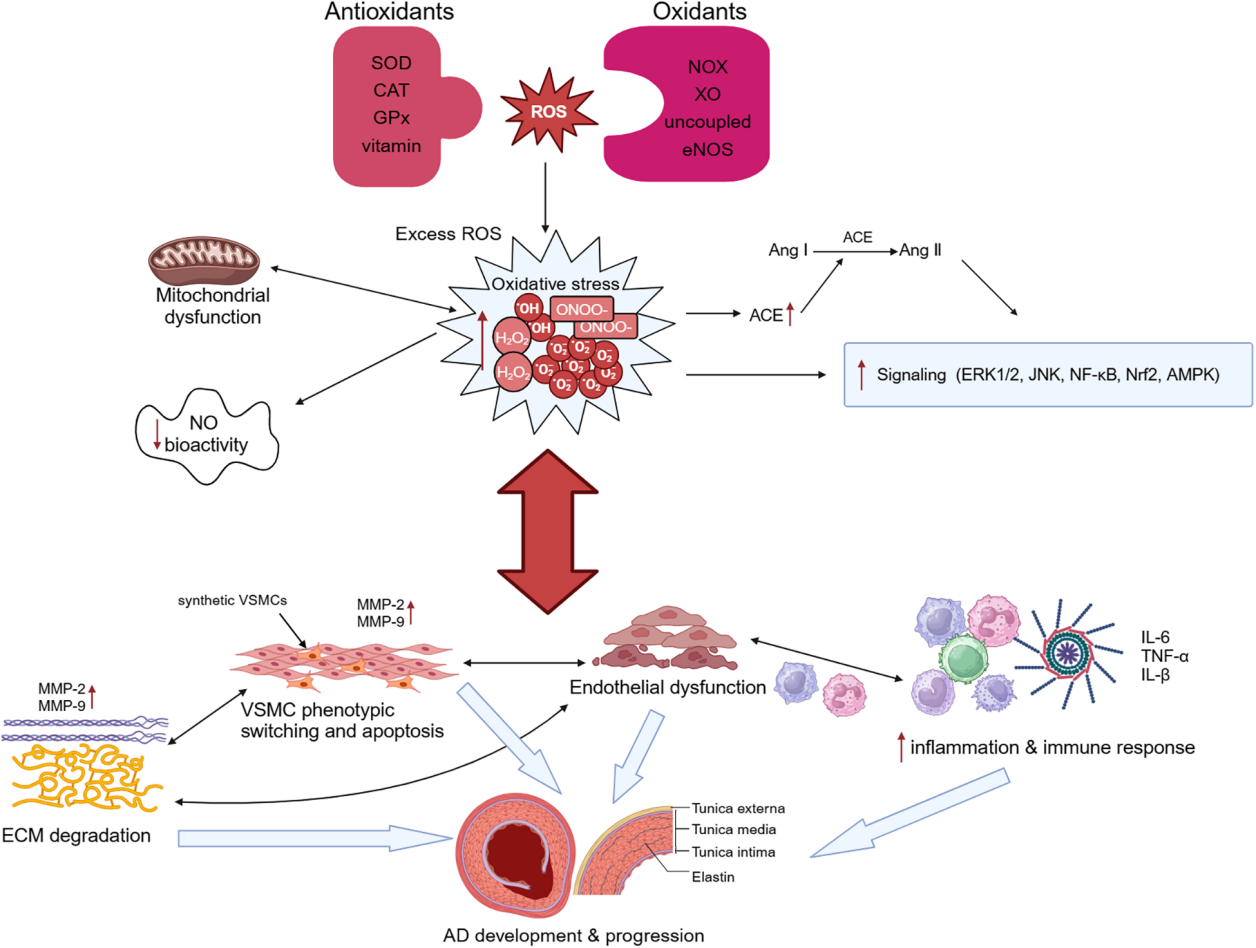
Oxidative stress and aortic dissection (AD). Overproduction of reactive oxygen species (ROS) and imbalance of intracellular antioxidant systems lead to oxidative stress. Oxidative stress causes mitochondrial dysfunction, which in turn exacerbates oxidative stress. Excessive ROS not only stimulates angiotensin-converting enzyme (ACE) expression and promotes the conversion of angiotensin I (Ang I) to Ang II, but also regulates the activation of many signaling pathways and reduces NO bioactivity. Oxidative stress can promote both phenotypic switching and apoptosis of vascular smooth muscle cells (VSMCs) and induce the degradation of extracellular matrix (ECM) by upregulating the expression of proteolytic enzymes such as matrix metalloproteinase (MMP)-2 and MMP-9. It additionally modulates the infiltration of macrophages and mononuclear lymphocytes and the secretion of cytokines, resulting in the disruption of endothelial cell structure and function, ultimately leading to endothelial dysfunction. These changes ultimately lead to the occurrence and development of AD. SOD, superoxide dismutase; CAT, catalase; GPx, glutathione peroxidase; NOX, triphosphopyridine nucleotide (NADPH) oxidase; XO, xanthine oxidase.

The key histological abnormalities of AD include necrosis and apoptosis of VSMCs, fracture of elastic fibers, and degradation of extracellular matrix (ECM) (73).The thrombi formed within the false lumen indicates the presence of continued inflammation under the condition of atherosclerosis and chronic inflammation, and the higher risk of rupture in patients with inflammatory diseases demonstrates the importance of inflammation in AD (1). Oxidative stress promotes pathological phenotypic switching and apoptosis of VSMCs, upregulates expression of proteolytic enzymes such as matrix metalloproteinases (MMPs), and induces the degradation of ECM (74). It also modulates proliferation of fibroblast and infiltration of macrophages and mononuclear lymphocytes, resulting in the disruption of endothelial cell structure and function, ultimately leading to endothelial dysfunction (75). All of those advances the pathological aortic wall remodeling and AD formation (Figure 2). Despite significant advances in our understanding of the molecular pathogenesis of AD over the past 20 years, therapeutic options to slow disease progression are still limited, and there are no preventive therapeutic options. Understanding the role of oxidative stress in AD can help us further explore its potential diagnostic, prognostic, and therapeutic value in AD.

### 4.1 Oxidative stress and vascular smooth muscle cells

The onset of AD is typically concurrent with the deterioration of contractility in VSMCs and the buildup of inflammatory mediators. The loss of VSMCs in the middle layer of aorta, including phenotypic switching and apoptosis, could represent a critical initial stage in AD formation (76). Following stimulation by various physiological factors (such as inflammatory triggers, vascular damage, and hemodynamic perturbations), contractile VSMCs in the normal aortic wall lose their unique structure and function, and undergo phenotypic switching to synthetic VSMCs, which is characterized by enhanced abilities of proliferation and migration, and increased secretion of various extracellular matrix proteins and cytokines (77). Studies have shown that ROS fosters vascular injury by promoting phenotypic switching and apoptosis of VSMCs (78, 79), while the inhibition of ROS reverses this effect (80).

Excessive production of ROS disrupts the oxidation/antioxidant equilibrium, instigates alterations in VSMC phenotype, and facilitates the initiation and advancement of AD. Oxidative stress stimulates angiotensin-converting enzyme (ACE) expression, which is responsible for the conversion of angiotensin I (Ang I) to Ang II. Ang II triggers mitochondrial dysfunction by activating NOX and generating NO, ONOO^−^, and $O_{2}^{\cdot -}$. NOX deficiency prevents Ang II-induced AD (81). NOX2 potentially modulates Ang II-induced vascular remodeling and hypertension through growth differentiation factor 6 Gene and other paracrine signaling factors in VSMCs (82). NOX1, serving as the primary NOX2 homolog in VSMCs, regulates VSMC growth and migration, whereas NOX4 controls VSMC phenotypic switching. Overexpression of smooth muscle cells-specific NOX1 enhances VSMC responsiveness to Ang II (83). Additionally, NOX1 governs Ca^2+^ signaling, contributing to blood vessel constriction and blood pressure regulation (84). Breast cancer susceptibility gene 1 shields VSMCs against oxidative stress by inhibiting NOX1-dependent ROS production (85). Furthermore, NOX1 impacts AD progression by negatively regulating fibrin-5 in VSMCs (86). NOX4 partly contributes to the development of Ang II-induced aortic aneurysms and atherosclerosis by modulating osteopontin expression (87). The cytochrome b558 membrane complex, comprised of NOX2 and p22phox, serves as the catalytic core responsible for generating $O_{2}^{\cdot -}$ through NOX. The p22phox regulates the activity of NOX4. Poldip2, a molecular chaperone for p22phox, can bind to p22phox, thereby activating NOX4, regulating VSMC phenotype and migration, and establishing a connection between ROS production and cytoskeletal remodeling (88). NOX4 and ROS are increased in the pericyte mediators of human Marfan aortic tissue and are transcriptionally overexpressed in VSMC. In Marfan mice, NOX4 deficiency results in delayed aortic aneurysm progression and normalization of endothelial dysfunction in Marfan aorta (89). Besides, NOX5 is a key regulator of the phenotypic switching of VSMCs, and Ca^2+^ can induce ROS production in VSMCs via NOX5 (90). Inhibiting NOX5 mitigates actin aggregation and migration in hypertensive VSMCs, thereby ameliorating hypertension-associated vascular damage (91). The transfer of the eNOS gene to VSMCs inhibits their proliferation (92, 93). Oxidative stress can induce VSMC proliferation and migration through the MAPK pathway. Alpha-ketoglutaric acid (AKG) is a key metabolite within the TCA cycle, exhibiting inhibitory effects on oxidative stress and inflammatory responses. AKG safeguards VSMCs by impeding the ERK1/2 pathway (94). SIRT1 overexpression amplifies oxidative stress through the MAPK signaling pathway, elevating cyclin expression and consequently fostering excessive VSMC proliferation (95). In human and mouse aneurysm tissues, vascular peroxidase 1 (VPO1) expression is upregulated and significantly intensified oxidative stress by catalyzing *H*_2_*O*_2_ to generate hypochlorous acid (HOCl). HOCl treatment triggers ERK1/2 phosphorylation and promotes VSMC phenotypic switching (96).

Increasing antioxidants and reducing ROS production can impede AD progression by inhibiting biological processes such as autophagy and apoptosis in VSMCs. CD38 serves as the primary degrading enzyme for nicotinamide adenine dinucleotide (NAD) in mammalian cells, an essential endogenous antioxidant in mammals. A study on Ang II-induced vascular remodeling in mice showed that CD38 deficiency leads to elevated NAD levels, mitigating Ang II-induced vascular aging through VSMC aging inhibition (97). Statins significantly enhance the DNA-binding activity of Nrf2 and induce the expression of its target genes (e.g., HO1 and GPx), thereby safeguarding endothelial cells and VSMCs from oxidative stress (98). Aldehyde dehydrogenase 2 (ALDH2), a mitochondrial enzyme, metabolizes major lipid peroxidation products. In the Chinese population, the ALDH2 rs671 is one of the most common functional genetic mutation loci. The activity of the ALDH2 enzyme significantly decreases owing to this mutation. ALDH2 rs671 polymorphism may expedite the onset and progression of cardiovascular diseases by modulating biological processes such as autophagy, apoptosis, and oxidative stress in cardiomyocytes and VSMCs through ROS-mediated lipid peroxidation (99). A study involving Ang II-induced aortic aneurysms in mice suggested that ALDH2 deficiency may enhance susceptibility to abdominal aortic aneurysm formation by diminishing anti-ROS effects and increasing VSMC apoptosis and vascular inflammation (100). However, a case-control study (total *n* = 706) conducted at two independent centers showed that ALDH2 deficiency inhibits VSMC phenotypic switching and reduces the risk of developing AD (101). This may imply the presence of a dose threshold for ALDH2 in regulating the risk of AD development.

### 4.2 Oxidative stress and ECM

A key histological hallmark of AD is the fragmentation of elastic fibers, disturbed collagen alignment and abnormal collagen deposition (102–104). Elastic and collagen fibers make up the fibrous network of the ECM. The ECM constitutes a dynamic network structure interconnecting cells and mediating cellular signal transduction. Irregularities in ECM synthesis and degradation can lead to a spectrum of diseases. Oxidative stress can perturb ECM component metabolism and influence the expression of related factors, resulting in excessive accumulation or degradation of specific ECM elements. Consequently, damage and diseases of tissue and organs can ensue (105). The mechanical characteristics of the aorta primarily hinge on elastic and collagen fibers. Elastic fibers, notably those affiliated with SMCs, are most abundant in the middle layer of the aortic wall. Two specific collagen types (Type I and Type III), serving as critical matrix components and the outer membrane enveloping fibers, confer tensile strength and sustain the structural integrity of blood vessel walls. This safeguard prevents arteries from undergoing excessive distention and rupturing (68, 106).

Fibroblasts in the outer membrane of the aorta play a role in ECM by regulating the production of type I collagen (107, 108). Myofibroblasts are specialized cells that have a more contractile phenotype and produce more ECM proteins than fibroblasts. Oxidative stress activates myofibroblasts and contributes to tissue repair and fibrotic remodeling. Overactivated myofibroblasts exhibit higher MMP activity and abnormal ECM synthesis capacity in AD, which may directly contribute to AD by producing disordered ECM and enhancing pro-inflammatory responses (108–110). MMPs and fibrinolytic enzymes degrade elastin and collagen (111–113). Both animal experiments and clinical investigations have substantiated the role of MMPs in AD initiation and progression (114–119). Ang II can promote the differentiation of aortic adventitial fibroblasts into myofibroblasts by inducing the phosphorylation of ERK1/2 and aggravate the formation of AD (120). Antagonizing Ang II signaling with losartan partially prevents stress-induced myofibroblast activation, collagen accumulation, intimal hyperplasia, and aortic dilatation (121).

Oxidative stress contributes to the progression of AD by upregulating MMPs, causing the degradation of the aortic middle layer and ECM remodeling. Numerous studies have shown that ROS can facilitate the degradation of elastin and collagen, induce VSMC apoptosis, and alter aortic compliance. This promotes the development of AD by activating MMP1 (122), MMP2 (123–125), MMP9 (126, 127) and other MMPs (128). MMP1 is a pivotal enzyme responsible for degrading both Type I and Type III collagen. SOD3 can mitigate intracellular ROS levels, suppress MMP1 expression, and preserve ECM integrity (122). MMP2 and MMP9 play roles in VSMC growth, proliferation, and migration. Ang II enhances the mRNA synthesis and activity of MMP2 in a P47PHOx-dependent, i.e., ROS-dependent, manner (129). Imbalances in the redox system can lead to the generation of oxidized LDL (ox-LDL), which activates NOX more than native LDL does. Ox-LDL also reduces activity of eNOS. MMP2 assumes a key role in ox-LDL-induced VSMC proliferation in both rabbits and humans (130). Oxidative stress-induced lipid peroxidation, along with its byproduct, 4-hydroxynonenal (4-HNE), contributes to the imbalance between elastin synthesis and degradation, thereby participating in vascular wall remodeling (131). Notably, 4-HNE can activate the mitochondrial ROS-mediated AKT/NF-κB signaling pathway and increase MMP2 production in VSMCs (123). MMP2 also possesses the capability to activate epidermal growth factor receptors, heighten ROS production, and stimulate vasoconstriction (124). Downregulating MMP2 serves to diminish elastin degradation, bolster eNOS activity, and delay vascular aging (125). Quercetin, a pivotal flavonoid with pronounced antioxidant properties, attenuates hypertension-induced aortic remodeling, oxidative stress, and MMP2 activity (132). Nitrite effectively impedes the activation of MMP2 via XO-mediated antioxidant mechanisms and reverses vascular structural alterations associated with hypertension (133). Adiponectin (APN) exerts a significant inhibitory effect on the proliferation and migration of VSMCs. MMP2 and MMP9 expression is diminished in mice pre-treated with APN, and APN hinders ROS-induced cardiomyocyte remodeling through AMPK activation, ERK signaling inhibition, and suppression of NF-κB activity (134). Insulin-like growth factor elicits ROS production through NOX4, heightening MMP2 and MMP9 activity, thereby promoting VSMC migration (135). Salusin-β fosters VSMC migration and induces vascular injury by augmenting MMP9 production via modulating NOX2 activation (127). In the context of non-enzymatic antioxidant systems, melatonin exhibits the capacity to forestall AD by modulating oxidative stress and VSMC proliferation. Moreover, melatonin treatment significantly curtails elastin degradation, macrophage infiltration, and MMP expression, and mitigates oxidative stress-induced damage (136). In conclusion, oxidative stress acts as a catalyst for deterioration of AD by augmenting MMP activation, thereby reshaping the ECM and aggravating degradation and remodeling of aortic wall.

### 4.3 Oxidative stress and inflammatory cells

Inflammation plays a crucial role in AD (137–141). Inflammatory cells, such as macrophages, exhibit a multifaceted impact by releasing ROS, upregulating the expression of MMPs and cell adhesion molecules, inducing ECM degradation and neovascularization, and fostering VSMC apoptosis, ultimately culminating in aortic cystic degeneration (142–144). A vicious cycle exists between oxidative stress and inflammation (145). Cytokines secreted by inflammatory cells inflict tissue damage, resulting in diminished blood vessel wall elasticity, eventually culminating in AD rupture. Concentrations of IL-6 (146), C-reactive protein (CRP), TNF-α, and IL-1β are significantly elevated in AD (147). Oxidative stress stimulates inflammatory cell infiltration directly and enhances cytokine secretion and inflammasome production of inflammatory cells, thereby amplifying the inflammatory response and exacerbating the initiation and progression of AD. Cytokines secreted by inflammatory cells can induce oxidative stress, promoting AD progression, and excessive ROS also augments cytokine secretion.

The progression of numerous inflammatory diseases is concomitant with the production of ROS, and excessive ROS can foster the infiltration of inflammatory cells. Oxidative stress is a pivotal mediator of Ang II signaling receptors in human neutrophils (148). In murine models of Ang II-induced AD, Ang II stimulates neutrophils via the angiotensin receptor AT1, leading to the induction of NOX, ROS production, and adhesion of mononuclear cells to endothelial cells (149). In response to this stimulation, neutrophil DNA is released into the extracellular space and facilitates the formation of extracellular traps known as NETs, which serve to immobilize and eradicate pathogens. The generation of NETs is coupled with neutrophil cell death, a unique mode distinct from apoptosis and cell necrosis, termed NETosis. Ang II-mediated NETosis relies on ROS and may contribute to aneurysmal vascular remodeling through elastin degradation (primarily involving MMPs) and collagen accumulation (involving tissue factors) (149). Classical activation of macrophages into the M1 phenotype induces tissue damage by releasing chemokines and potent oxidants. Elevated ROS levels induce macrophages to polarize toward the M1 phenotype, thereby augmenting the release of inflammatory cytokines and promoting a pro-inflammatory role (150). Kinases Mst1 and Mst2 detect ROS and uphold the redox equilibrium within macrophages by modulating the stability of the antioxidant transcription factor Nrf2 (151). Atherosclerosis is a notable risk factor for AD. Inflammatory cells augment ROS and ox-LDL production by secreting oxidase, thereby compromising the functionality of endothelial cells and VSMCs within the vascular system. Macrophages avidly ingest ox-LDL in substantial quantities and eventually form foam cells through scavenger receptors such as CD36 and lectin-like ox-LDL receptor 1 (LOX-1) (152). Ox-LDL reconfigures the fatty acid metabolism within macrophages and sustains chronic inflammation by upregulating CD36 and its downstream fatty acid transport system (153). LOX-1 inhibits macrophage migration and instigates foam cell formation, and then promote the production of MMP9 (154). Oxidative stress can induce mitochondrial dysfunction in macrophages, facilitate conversion of macrophages to foam cells, and induce a pathological switching in the VSMC phenotype. Mitochondrial oxidative stress in macrophages contributes to the progression of atherosclerosis by enhancing the production of monocyte chemotaxis protein-1 and other inflammatory processes through mediating the NF-κB signaling pathway (155).

IL-6 induces oxidative stress and endothelial dysfunction by upregulating Ang II Type 1 receptors (156). Carbon monoxide-releasing molecule 2, a CO-releasing pharmacological donor, mitigates Ang II-induced VSMC migration by inhibiting ROS production and reducing IL-6 and MMP9 increments (157). A positive correlation exists between CRP concentration and oxidative stress levels, and activated neutrophils can induce oxidative stress by releasing ROS and pro-inflammatory cytokines (158). TNF-α inhibition ameliorates hypertensive vascular hypertrophy by reducing MMP2 activity and ROS production (159). IL-1β amplifies ROS production and fosters VSMC migration, proliferation, and endothelial dysfunction via the TLR4 pathway. Nrf2 activation inhibits IL-1β and interferes with the TLR4 pathway, safeguarding blood vessels from damage (160). Sestrin2, primarily secreted by macrophages, suppresses ROS production, affording cellular protection against various noxious stimuli. Sestrin2 plays a protective role in AD by mitigating Ang II-induced VSMC cell apoptosis through the Nrf2 pathway (161).

The inflammasome is a complex of multiple proteins that serve as cytoplasmic receptors within the innate immune system. They possess the ability to detect pathogens and trigger inflammatory responses, both in normal and pathological conditions. Among these, the NLRP3 inflammasome stands out as the most extensively investigated, chiefly responsible for the secretion of bioactive IL-1β and IL-18, consequently inducing inflammatory cell demise (162). ROS is a catalyst for activation of NLRP3 inflammasome, thereby recruiting macrophages and neutrophils (163). The NLRP3 inflammasome exerts crucial effects in the initial inflammatory reaction associated with AD (164). The inflammasome within macrophages exacerbates the advancement of AD, while the absence of NLRP3 mitigates oxidative stress and regulates macrophage polarization. The suppression of mitochondrial ROS generation in macrophages effectively curtails inflammasome activation (165). Activation of Nrf2 can prevent BAPN-induced AD by impeding ROS-mediated activation of the NLRP3 inflammasome, concurrently diminishing the invasion of macrophage and production of MMPs and pro-inflammatory cytokines (166).

### 4.4 Oxidative stress and endothelial cells

Vascular endothelial cells not only function as a selective barrier but also synthesize and secrete diverse active molecules, participate in inflammatory processes, and regulate vascular elasticity. These functions are important in governing blood vessel elasticity and configuration (167). Oxidative stress-induced endothelial dysfunction is believed to play a significant role in the development of AD (168). In mouse models of Ang II infusion-induced AD, augmented production of specific ROS within the endothelial cells of NOX2 transgenic mice was found to contribute to Ang II-mediated AD (11).

As a crucial rate-limiting enzyme of NO production in endothelial cells, eNOS assumes a pivotal role in the occurrence and development of AD. Oxidative stress can induce eNOS dysfunction and, consequently, impair vascular endothelial function. Mice with both eNOS and apolipoprotein E gene knockouts exposed to a Western diet for a 24-week duration exhibit the spontaneous development of abdominal aortic aneurysms and AD (169). Endothelial cells produce excess ROS through mitochondria, NOX, and XO, which not only lead to the clearance of NO but also induce eNOS uncoupling, thereby exacerbating oxidative stress (170). eNOS consists of two subunits connected by a zinc finger structure and each of subunit comprises two structural regions: an oxidation region and a reduction region. The oxidation region encompasses binding sites for BH_4_, L-Arg, and heme, while the reduction region contains binding sites for flavin adenine dinucleotide (FAD), flavin mononucleotide (FMN), and NADPH. The junction between the oxidation and reduction regions is where calmodulin (CaM) binds. When CaM from two molecules combines, eNOS becomes activated and leads to the conversion of *O*_2_ to NO and L-Arg to L-citrulline (170).

**Figure 3:**
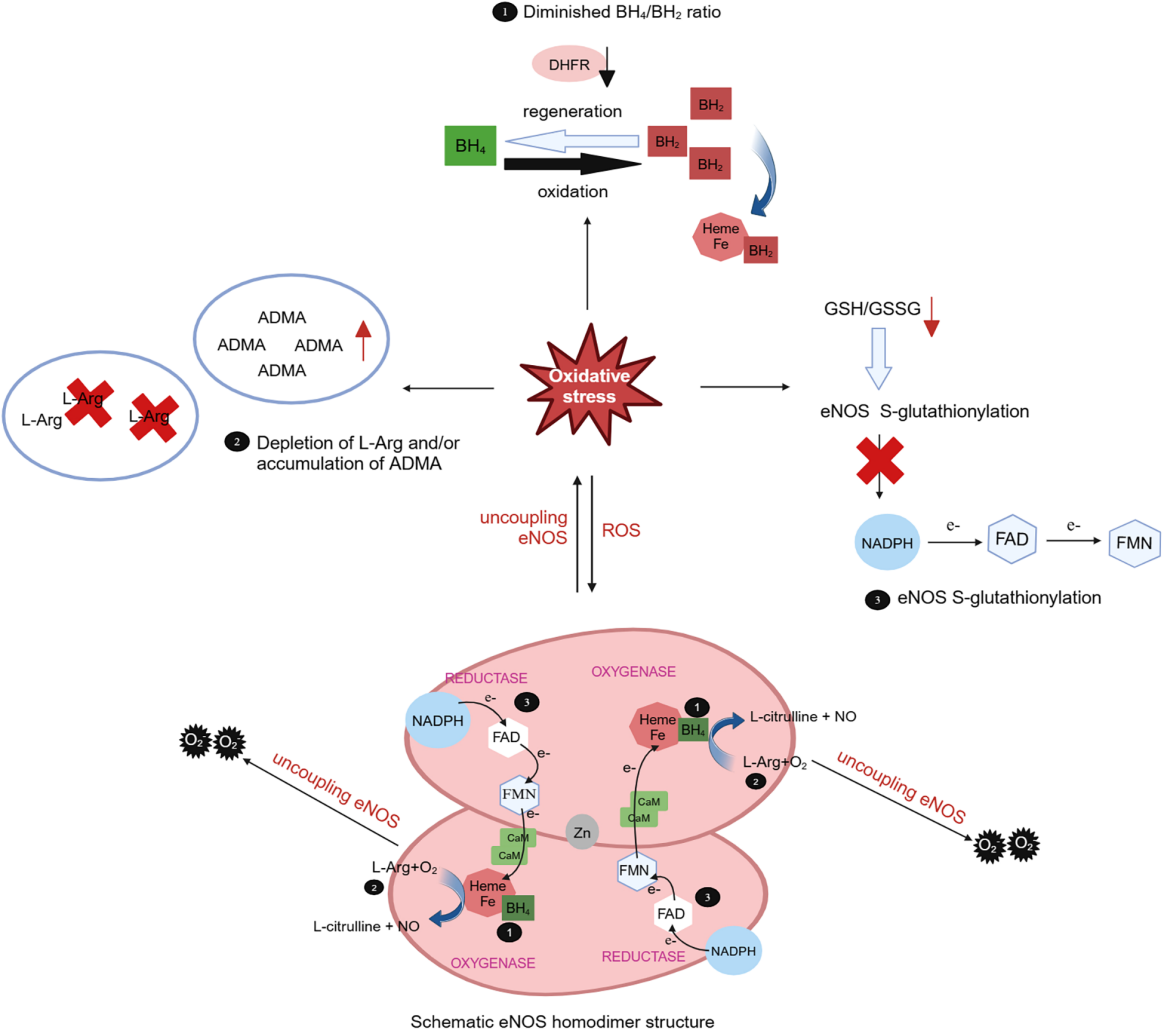
Role of oxidative stress in eNOS uncoupling. The eNOS utilizes L-arginine (L-Arg) and oxygen as substrates to synthesize NO and L-citrulline with the indispensable cofactor tetrahydrobiopterin (BH4). Meantime, the triphosphopyridine nucleotide (NADPH) was oxidated and electrons were transferred to heme by flavin adenine dinucleotide (FAD) and flavin mononucleotide (FMN). In cases of eNOS uncoupling, $O_{2}^{\cdot -}$ is produced instead of NO. The role of oxidative stress in eNOS uncoupling due to oxidative depletion of tetrahydrobiopterin (BH_4_), deficiency of the eNOS substrate L-Arg or accumulation of asymmetric dimethylarginine (ADMA), and eNOS S-glutathionylation. Under oxidative stress, BH_4_ is rapidly oxidized by superoxide anion to BH_2_, which competitively replaces BH_4_ for heme binding and promotes eNOS uncoupling. BH2 can subsequently be regenerated into BH4 through the dihydrofolate reductase (DHFR). Oxidative stress can lead to a decrease in the L-Arg/ADMA ratio, effectively reducing substrate utilization. Oxidative stress also decreases the intracellular glutathione (GSH)/oxidized glutathione (GSSG) ratio, leading to eNOS S-glutathionylation and impede electron transport.

The oxidative depletion of the eNOS cofactor BH_4_, deficiency in the eNOS substrate L-Arg or its analogue, accumulation of asymmetric dimethylarginine (ADMA) can result in eNOS uncoupling, leading to endothelial dysfunction and ultimately contributing to cardiovascular disease. Oxidative stress plays a pivotal role in all three mechanisms (170). BH_4_ serves as a key cofactor for eNOS, exerting substantial influence over eNOS activity. Under oxidative stress conditions, BH_4_ is swiftly oxidized to BH_2_ by superoxide anions, particularly peroxynitrite generated by the scavenging of NO by *O*_2_, and BH_2_ can subsequently be regenerated into BH_4_ through the dihydrofolate reductase (DHFR) recovery pathway. BH_2_ can competitively displace BH_4_ in its binding to heme, thereby promoting eNOS decoupling (171) (Figure 3). In cardiovascular diseases, oxidative stress not only accelerates the oxidative depletion of BH_4_ but also affects the expression and activity of DHFR (170). Ang II downregulates DHFR, inducing BH_4_ deficiency in an *H*_2_*O*_2_-dependent manner. Overexpression of DHFR can restore NO production and reduce the $O_{2}^{\cdot -}$ generated by eNOS (172). Oral folic acid supplementation or overexpression of DHFR has been shown to prevent aneurysm formation in mice with Ang II-induced AD by effectively mitigating eNOS uncoupling and attenuating vascular remodeling and inflammation. This includes the reduction of medial elastin breakdown, MMP2 and MMP9 activation, and macrophages infiltration (173). eNOS uncoupling may manifest activation of downstream NOX. In double knockout mice lacking the NOX1, NOX2, or NOX4 genes alongside the BH_4_ gene, respectively, the bioavailability of NO and BH_4_ in endothelial cells is increased, DHFR content is significantly increased, and the incidence of abdominal aortic aneurysms is significantly decreased, as compared with mice with knockout of the BH_4_ gene only. This suggests that inhibiting the NOX signaling pathway can decelerate aneurysm progression by mitigating oxidative stress-induced damage in endothelial cells (174). These findings underscore the tight linkage between oxidative stress in endothelial cells and the development of AD.

In the development of AD, VSMCs, ECM, inflammatory cells, and endothelial cells engage in intricate interactions. Endothelial cells exert influence over the proliferation, migration, and phenotypic switching of VSMCs through the secretion of vasoactive substances, thereby altering the configuration of the blood vessel walls. Vascular endothelial cells secrete not only vasodilatory substances such as NO but also other active compounds, including endothelin 1 (ET1) and transforming growth factor-β (TGF-β). NO can bind to the heme moiety of VSMCs, activating guanylate cyclase to elevate cyclic guanosine phosphate (cGMP) levels. Subsequently, cGMP becomes a secondary messenger that inhibits the proliferation of VSMCs and promotes apoptosis of VSMCs (175). The NO/eNOS signaling pathway plays a crucial role as a negative regulator of VSMC growth. Impairment of NO synthesis in endothelial cells due to oxidative stress results in significant proliferation of VSMCs in the aortic walls (176). The overexpression of ET1 in endothelial cells induces vascular remodeling and endothelial dysfunction by activating NOX (177). ROS mediates the activation of ET1-induced ERK1/2 signaling and protein synthesis in VSMCs (178). The equilibrium between NO and ET1 governs the apoptosis and survival of VSMCs, respectively. ET1 inhibits NO-induced apoptosis of VSMCs through the MAPK pathway (179). Oxidative stress can activate multiple signaling pathways in endothelial cells and augment TGF-β secretion (180). When co-cultured with endothelial cells, endothelial-derived TGF-β can induce the switching of VSMCs into a secretory phenotype by regulating the phosphoinositide 3-kinase/AKT signaling pathway (181, 182). Endothelial cell dysfunction results in ECM metabolism disorders, VSMC apoptosis, aortic wall thinning, and then aortic aneurysms and dissection. The NF-κB signaling pathway mediates the upregulation of adhesion molecules in endothelial cells. In mice, specifically blocking the NF-κB pathway in endothelial cells significantly reduces the expression of adhesion molecules and inflammatory factors, macrophage infiltration, matrix degradation, and oxidative stress in endothelial cells. This ultimately reduces inflammation and artery dilation in mice (183). NLRP3 inflammasome activation exacerbates oxidative stress and endothelial dysfunction, while inhibition of the NLRP3 inflammasome signaling pathway can ameliorate vascular dysfunction (184).

## 5 AD treatment

### 5.1 Therapies targeting the enzyme systems that generate ROS

**Figure 4:**
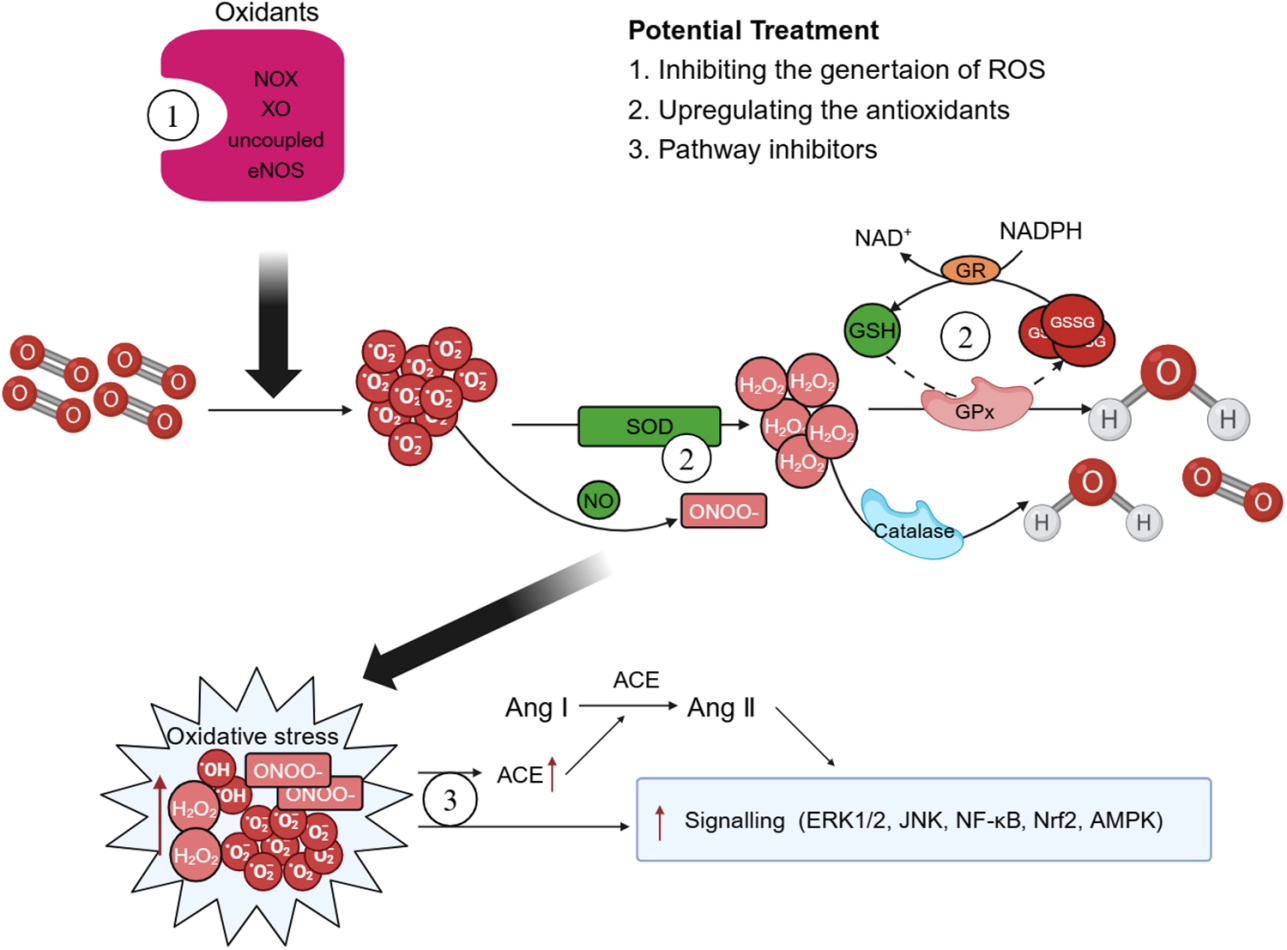
Possible therapeutic targets. There are some potential therapeutic medicines that associated with oxidative stress. Therapies targeting the enzyme systems that generate oxidative stress and reactive oxygen species (ROS), such as triphosphopyridine nucleotide (NADPH) oxidase (NOX) inhibitors may play a crucial role in the improve of aortic dissection (AD). Superoxide dismutase (SOD) mimics and polyphenols can enhance the body's antioxidant capacity and exert effects of reduce oxidative stress. Moreover, ROS exerts multiple effects on the development of AD pathology by regulating different signaling pathways and treatments associated with the activation of ROS-mediated signaling pathways should also be explored more closely. GPx, glutathione peroxidase; GSH, glutathione; GSSG, oxidized glutathione; GR, glutathione reductase; XO, xanthine oxidase; ACE, angiotensin-converting enzyme; Ang, angiotensin.

In recent years, both preclinical and clinical studies have elucidated several endogenous enzyme systems that regulate oxidative stress in AD. Given the critical role played by NOX in AD, there is potential for selectively targeting specific NOX subtypes to rectify eNOS uncoupling and mitochondrial dysfunction, thereby offering a viable approach for the prevention and treatment of AD (15). Additionally, SOD mimics can also be used to enhance the effect of SOD, remove harmful $O_{2}^{\cdot -}$ from the body, diminish the infiltration of inflammatory cells, and retard the aging process (185). Polyphenols, which are natural antioxidants, inhibit the production of MMPs and thwart ROS-induced signal transduction (186). Polyphenols mitigate the necrosis and apoptosis in myocardial cells, decrease the infarct size of heart, and enhance cardiac function by reducing ROS or reactive nitrogen-free radical production stemming from oxidative stress (187) (Figure 4).

### 5.2 Treatments associated with the ROS-mediated signaling pathways

ROS exerts multiple effects on the development of AD pathology by regulating different signaling pathways. Calcium channel blockers such as nifedipine can inhibit the activation of the MAPK signaling pathway to regulate inflammation (188). Lipid-regulating drugs also exhibit anti-inflammatory effects by inhibiting MAPK activity and maintaining endothelial cell junctional homeostasis (189). Drugs acting on the renin-angiotensin system are involved in the NF-κB and MAPK signaling pathways and reduce the expression of inflammatory factors such as IL-4 and TNF-α. LMA, a major component of codonopsis lanceolata, displays antioxidant effects not only by downregulating ROS production via inhibiting expression/phosphorylation of NOX subunits but also by inhibiting the activation of JNK and NF-κB pathway (190). Additionally, Senkyunolide I (SEI) is a traditional Chinese medicine with notable anti-inflammatory functions. SEI can improve the progression of AD through suppressing the NF-κB activity, and thereby attenuating the production of inflammatory cytokines, oxidative stress, and apoptosis in endothelial cells (191).

### 5.3 Supplementing the endogenous and exogenous antioxidants

The primary endogenous antioxidants in mammalian cells include NAD, GSH, and vitamin C, which collectively shield cells from oxidative stress. The supplementation of NAD or GSH precursors improves cardiac function and redox status in models of heart failure (192–195). NAD precursors include nicotinamide (196), nicotinamide ribose (197), nicotinamide mononucleotide, and nicotinic acid (198). Augmenting NAD levels can extend a healthy lifespan, mitigate metabolic syndrome, and reduce blood pressure (198). GSH is synthesized through the gamma-glutamyl cycle and procysteine has demonstrated the capacity to elevate endogenous GSH production, rendering it a promising area of research in cardiovascular diseases (8). Vitamin C serves to safeguard BH4, reinstating eNOS enzyme functionality and enhancing aortic endothelial performance (199, 200). Moreover, it curbs ROS production and amplifies NO availability in endothelial cells, thus averting acute alcohol-induced endothelial dysfunction (201). Furthermore, aside from bolstering endogenous antioxidant capabilities, an alternate approach to mitigating oxidative stress involves supplementing with exogenous antioxidants. As a natural polyphenolic antioxidant, resveratrol has abilities for anti-inflammatory and cardiovascular protection. In murine models of AD, resveratrol prevents vascular senescence by inhibiting NOX activity and reducing oxidative stress in a SIRT1-dependent way (202).

## 6 Limitations and prospects

Increased oxidative stress is regarded as a potential common etiological factor in diverse cardiovascular diseases. Extensive research has explored the role of oxidative stress in the initiation and progression of AD. There is substantial evidence indicating the involvement of ROS in the pathophysiology of AD. However, the pathogenesis of AD is intricate, and no solitary mechanism can comprehensively elucidate its pathophysiological processes. Oxidative stress and inflammation are acknowledged as major contributing factors. Our endeavor was to elucidate the role of oxidative stress in the occurrence and advancement of AD through a comprehensive analysis of existing studies. This analysis encompassed the impact of oxidative stress on VSMCs, ECM, inflammatory cells, and endothelial cells. Encouraging outcomes from preclinical investigations have showcased the potential efficacy of diverse antioxidant strategies.

As a result of these very promising results in animal models, several studies have evaluated the potential of anti-oxidative stress therapies in clinical settings (203, 204). However, clinical trials of various antioxidants have shown no benefit (205). In fact, the evidence from recent intervention studies of high-dose antioxidant vitamins and other antioxidants in food has been very disappointing (206, 207). Although targeting oxidative stress is theoretically logical, most strategies currently employed in the clinical setting have failed to improve patient prognosis. The exact reasons and mechanisms regarding the failure of these studies to produce the expected beneficial effects remain largely unknown. Still, we should not completely ignore studies targeting oxidative stress, especially endogenous antioxidant capacity, in AD patients. It is noteworthy that NAC supplementation to increase endogenous GSH levels and thus antioxidant capacity resulted in improved prognosis in patients with heart failure without any adverse side effects. Future oxidative stress therapies should focus on increasing endogenous antioxidant capacity rather than inhibiting oxidative stress production or supplementing with exogenous antioxidants (8).

Although these favorable effects observed in clinical trials did not consistently translate into positive patient outcomes, they did manifest as a reduction in cardiovascular and all-cause mortality. Considering these observations, the pursuit of novel therapeutic targets for mitigating oxidative stress emerges as a promising avenue for advancing the treatment of AD.
